# Feedback dynamic control for exiting a debt-induced spiral in a deterministic Keen model

**DOI:** 10.1371/journal.pone.0295859

**Published:** 2024-02-09

**Authors:** Ivan Perez Avellaneda, Francisco Rosales, Luis A. Duffaut Espinosa

**Affiliations:** 1 Department of Electrical and Biomedical Engineering, University of Vermont, Burlington, Vermont, United States of America; 2 Graduate School of Business, Universidad ESAN, Lima, Peru; Institute of Space Technology, PAKISTAN

## Abstract

The Keen model is designed to represent an economy as a dynamic system governed by the interactions between private debt, wage share, and employment rate. When certain conditions are met, the model can lead to a debt spiral, which accurately mimics the impact of a financial crisis on an economy. This manuscript presents a recipe for breaking this spiral by expressing Keen’s model as an affine nonlinear system that can be modified through policy interventions. We begin by considering critical initial conditions that resemble a financial crisis to achieve this goal. We then locate a desired point within the system’s vector field that leads to a desirable equilibrium and design a path towards it. This path is later followed using one-step-ahead optimal control. We illustrate our approach by presenting simulated control scenarios.

## 1 Introduction

The global financial crisis of 2008 stands as a significant event in economic history. It was sparked by the collapse of the US subprime mortgage market, leading to severe repercussions across the world economy. Researchers have extensively analyzed this and other crises in financial markets [1–3], providing valuable insights into their causes and consequences. According to studies by Reinhart and Rogoff [4], one of the underlying reasons of the crisis was the rapid accumulation of excessive household debt, fueled by lax lending standards and financial deregulation. Minsky [5, 6], had previously warned about the inherent instability of financial systems and the tendency for economic cycles to swing from periods of exuberance to crises [7–9]. This concept, known as the “financial instability hypothesis” (FIH), resonates with the dynamics observed during the crisis, which ultimately led the economy to a debt spiral with high unemployment and low wages.

Re-directing the economy to a stable equilibrium can be seen as an engineering control problem in a dynamical system [10–12]. The specialized literature [13–17] has shown the importance of incorporating control theory in economic and financial analysis, highlighting its potential to enhance policy effectiveness and mitigate economic fluctuations [18, 19]. Furthermore, the application of control techniques from different fields [20–24] has a long tradition in macroeconomics [25, 26], where models incorporating feedback control [27] have been employed to address issues such as inflation control [28], and monetary policy implementation [29]. Moreover, control techniques have found utility in the study of chaotic financial systems [30, 31], and in applications for risk assessment, portfolio optimization, and asset pricing, see e.g. [32]. For example, research by Zheng et al. [33] has demonstrated the benefits of using control theory to develop robust investment strategies that adapt to changing market conditions and enhance financial performance; while the works of [34, 35] have shown the use of control engineering to manage private and public debt respectively.

In this document, we follow Minsky’s FIH and use Keen’s model [36, 37] to characterize the economic system. This model emphasizes the interplay between debt, asset prices, and firm behavior that occurs during a financial crisis, mimicking the non-linear cyclical behavior of the economy. Our main objective is to apply engineering control techniques to the Keen model and present a recipe to break the debt spiral by expressing it as an affine nonlinear system that can be modified through policy interventions. We begin by considering critical initial conditions that resemble a financial crisis to achieve this goal. We then locate a desired point within the system’s vector field that leads to a desirable equilibrium and design a path towards it. This path is later followed using one-step-ahead optimal control, which situates this study in the realm of dynamically controlled systems [38, 39].

We have formalized our findings in the form of propositions and theorems to summarize our ideas and justify our proposals. Our main result (see theorem 3.1 in section 3) shows that there exists a unique control law for the Keen model. This result allows us to present a roadmap to exit the debt spiral characterizing the financial crisis of 2008. We stress that the utilized control policies require the crucial assumption that certain parameters in the Keen model can be altered or “controlled” under a certain level of latency. Namely, we consider that it is possible to control real interest rates with almost no latency, as its level is a decision made by the Central Bank; but adding extra dynamics to allow for a slow response when controlling the growth rate of labor productivity and the growth rate of the labor force, which, if controllable, can only be assumed to change at a much slower rate. To the best of our knowledge, this represents the pioneering integration of control engineering techniques into the Keen model. Consequently, this research augments the theoretical and empirical discourse surrounding this specific financial system in academic literature.

The rest of the manuscript is organized as follows. In section 2, the Keen model is presented. In section 3 the Keen model is revisited and is presented in the form of a non-linear affine system and the conditions of its controllability are discussed. In section 4, a simulation study is performed to explore the behavior of the system under different stress scenarios. Finally, Section 5 provides a discussion of the results and the conclusions resulting from this research. To make this a self-contained document, we added a technical appendix summarizing the basic ideas of linear control systems and feedback linearization that utilized in this manuscript.

## 2 Modeling financial instability

A state-space control model represents a dynamical system in terms of its states, inputs, and outputs. In economic terminology, the states are known as endogenous variables, while the inputs are referred to as exogenous variables. The outputs, on the other hand, do not have a specific name within economics, but they can be considered as performance variables that relate to a predetermined target for the system. For example, in the context of the money market, the Central Bank aims to decrease inflation (output) by increasing endogenous interest rates (state) via the reduction of exogenous money supply (input).

In this section, we present the Keen model [36] as an appropriate representation of the economy that enables us to demonstrate a debt share control mechanism in the system’s state space. We use this terminology to describe a procedure for guiding the current state of the economy from an initially unstable region to a desired location in a stable region. While exact tracking of the reference trajectory is not our primary concern, it is essential that the system states are not significantly distant from such a reference and do not violate the positivity of the interest rate.

### 2.1 Goodwin Model

**Fig 1:**
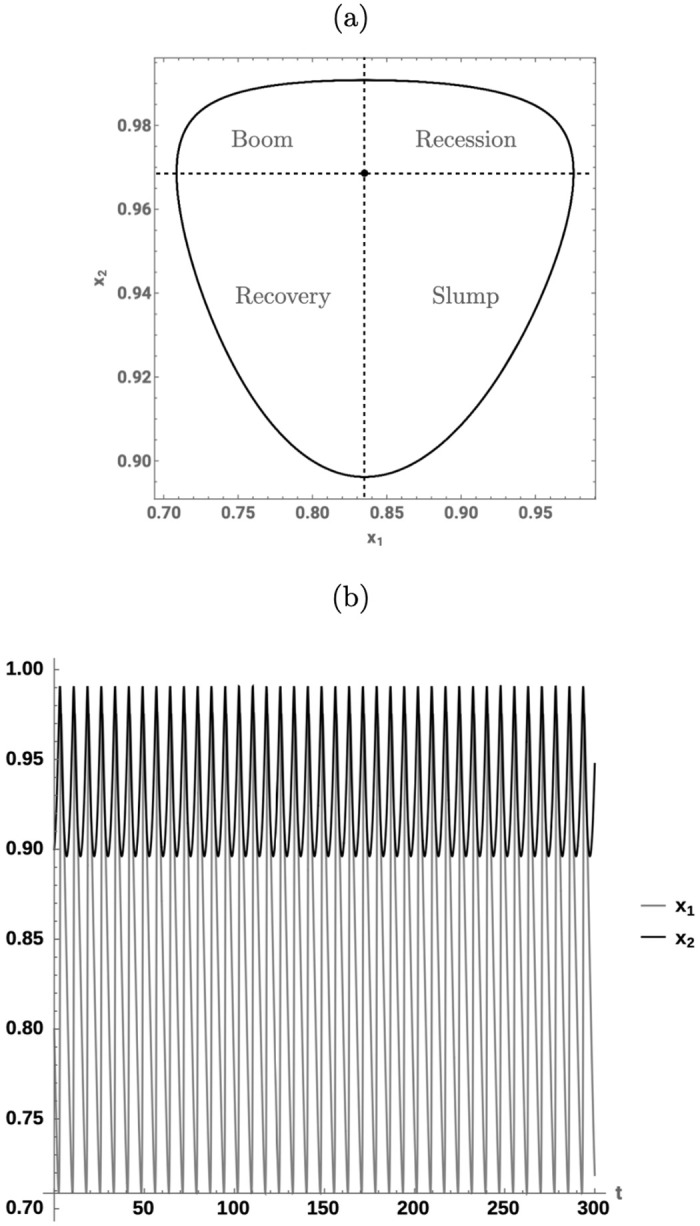
Goodwin model. (a) Phase portrait trajectory of the Goodwin model for the parameters in Table 1. The first quadrant is divided into four regions according to the growth and acceleration of wage share (*x*_1_) and employment rate (*x*_2_), around the stable non-trivial equilibrium given at (0.835, 0.969). (b) Simulated trajectories of wage share (*x*_1_) and employment rate (*x*_2_) for the system in (a).

Consider the Goodwin model [40] as a representation of an economy, resulting from the interaction between the wage share (*x*_1_) and the employment rate (*x*_2_), governed by the following system:

$$\begin{array}{c} {\dot{x}}_{1}=x_{1}[\Phi (x_{2})-\alpha ], \end{array}$$ (1a)

$$\begin{array}{c} {\dot{x}}_{2}=x_{2}[\frac{1-x_{1}}{\nu }-\alpha -\beta -\lambda ], \end{array}$$ (1b)

where *α* is the rate of growth of labor productivity, *β* is the rate of growth of labor force, λ is the capital depreciation constant, *ν* represents the capital-to-output ratio, and $\Phi \left(x\right)≔\frac{\phi_{1}}{{\left(1-x\right)}^{2}}-\phi_{0}$, denotes the Phillips curve for constants *ϕ*_0_, *ϕ*_1_ > 0. In addition, note that the state variables *x* = (*x*_1_, *x*_2_)^⊤^ take values in the set (0, 1) × (0, 1), where (0, 1) denotes the open interval between 0 and 1. Enforcing the condition

$$\begin{array}{c} \Phi^{-1}\left(\alpha \right)\Phi^{\prime }\left({\bar{x}}_{2}\right)\left(1-\nu \left(\alpha +\beta +\lambda \right)\right)>0, \end{array}$$ (2)

it is clear that (1) has a unique non-hyperbolic stable equilibrium

$$\begin{array}{c} \left({\bar{x}}_{1},{\bar{x}}_{2}\right)=\left(1-\nu \left(\alpha +\beta +\lambda \right),\Phi^{-1}\left(\alpha \right)\right), \end{array}$$ (3)

and its solutions are given by periodic curves centered at (3). Fig 1 shows the phase portrait of the system around (3) and illustrates the endogenous cycle of the system. We note that the Goodwin model also allows for an unstable equilibrium at (0, 0) and that this point is a saddle point if: i) Φ(0) − *α* > 0 and ii) $\frac{1}{\nu }-\alpha -\beta -\lambda <0$. However, we do not study this equilibrium further as it is of no economic interest.

Model (1) can also be studied from a control perspective. We call a system *controllable* if there exists a policy, in the form of inputs to the dynamical system, that can move it from an initial state to a final one, see e.g. [38, 39]. Here, we will briefly assess the controllability of the Goodwin model for inputs *u*_1_ ≔ *α* and *u*_2_ ≔ *β*, in terms of the system’s Taylor linearization and a weaker form of controllability known as *accessibility* (see Appendix A). We stress that our intention is to provide the necessary arguments to illustrate that (1) can indeed be manipulated, see e.g., [41, 42], and not to present a complete treatment of the theory of controllability of dynamical systems.

For a Taylor linearization procedure, let ***x***_0_ = (*x*_01_, *x*_02_)^⊤^ and ***u***_0_ = (*u*_01_, *u*_02_)^⊤^ give nominal points around which a Taylor expansion of (1) can be performed, such that ***x*** ≈ ***x***_0_ + *δ****x*** and ***u*** ≈ ***u***_0_ + *δ****u***. One thus has the linearized dynamics

$$\begin{array}{c} \dot{\delta x}=A\delta x+B\delta u, \end{array}$$

where

$$A=\left[\begin{array}{cc} \frac{\phi_{1}}{{\left(1-\text{x2}\right)}^{2}}-\phi_{0} & \frac{2x_{1}\phi_{1}}{{\left(1-x_{2}\right)}^{3}}\\ -\frac{x_{2}}{\nu } & \frac{1-x_{1}}{\nu }-\lambda \end{array}\right]{|}_{\begin{array}{l} x=x_{0}\\ u=u_{0} \end{array}},\;B=\left[\begin{array}{cc} -x_{1} & 0\\ -x_{1} & -x_{2} \end{array}\right]{|}_{\begin{array}{l} x=x_{0}\\ u=u_{0} \end{array}}$$

Therefore, from (27), one has that

$$\begin{array}{cc} C_{A,B} & =[B,AB]\\ & =[\begin{array}{cccc} -x_{01} & 0 & -\frac{2x_{01}^{2}\phi_{1}}{{\left(1-x_{02}\right)}^{3}}-x_{01}(\frac{\phi_{1}}{{\left(1-x_{02}\right)}^{2}}-\phi_{0}) & -\frac{2x_{01}x_{02}\phi_{1}}{{\left(1-x_{02}\right)}^{3}}\\ -x_{01} & -x_{02} & \frac{x_{01}x_{02}}{\nu }-x_{01}(\frac{1-x_{01}}{\nu }-\lambda ) & -x_{02}(\frac{1-x_{01}}{\nu }-\lambda ) \end{array}], \end{array}$$

where it is clear that $\text{Rank}(C_{A,B})=2$ for any ***x***_0_ ∈ (0, 1) × (0, 1), which ensures the existence of a local input policy ***u*** = (*u*_1_, *u*_2_)^⊤^ that can manipulate (1) towards a desired state. More generally, one can check the accessibility of (1) (see Appendix A), which we present in the form of the next proposition.

**Proposition 2.1**. *The Goodwin model described in* (1) *is accessible for all states* ***x*** *in the set* (0, 1) × (0, 1).

*Proof*. Showing that (3) is accessible follows from computing (32) and checking that its rank is 2. That is, from (1), one can see that

$$\begin{array}{cc} Q(f,g) & =[\begin{array}{ccccc} f & g_{1} & g_{2} & \left[f,g_{1}\right] & \left[f,g_{2}\right] \end{array}]\\ & =[\begin{array}{ccccc} x_{1}(\frac{\phi_{1}}{{\left(1-x_{2}\right)}^{2}}-\phi_{0}) & -x_{1} & 0 & \frac{2x_{1}x_{2}\phi_{1}}{{\left(1-x_{2}\right)}^{3}} & \frac{2x_{1}^{2}\phi_{1}}{{\left(1-x_{2}\right)}^{3}}\\ x_{2}(\frac{1-x_{1}}{\nu }-\lambda ) & -x_{2} & -x_{1} & -\frac{x_{1}x_{2}}{\nu } & x_{1}(\frac{\phi_{1}}{{\left(1-x_{2}\right)}^{2}}-\phi_{0})+x_{1}(\frac{1-x_{1}}{\nu }-\lambda ) \end{array}], \end{array}$$

where it is clear that Rank(*Q*(*f*, *g*)) = 2 for all ***x*** ∈ (0, 1) × (0, 1). Hence the system is locally accessible in (0, 1) × (0, 1), which is the region of interest for the control of the Goodwin model, and thus it is accessible.

### 2.2 Keen model

The Keen model can be seen as a direct extension of the Goodwin model in the following differential system.

$$\begin{array}{c} {\dot{x}}_{1}=x_{1}[\Phi (x_{2})-\alpha ], \end{array}$$ (4a)

$$\begin{array}{c} {\dot{x}}_{2}=x_{2}[\frac{\kappa (x_{1},x_{3})}{\nu }-\alpha -\beta -\lambda ], \end{array}$$ (4b)

$$\begin{array}{c} {\dot{x}}_{3}=x_{3}[r-\frac{\kappa (x_{1},x_{3})}{\nu }+\lambda ]+[\kappa \left(x_{1},x_{3}\right)-\left(1-x_{1}\right)], \end{array}$$ (4c)

where *x*_3_ represents the economy’s debt share, *r* is the debt’s real interest rate, and *κ* is a private investment function. Note that the first two equations in (4) are identical to those in (1) for the appropriate choice of *κ*(⋅). In (4), we define this function as *κ*(*x*_1_, *x*_3_) ≔ *κ*_0_ + *κ*_1_ tan^−1^(*κ*_2_(1 − *x*_1_ − *rx*_3_) + *κ*_3_), for constants *κ*_0_, *κ*_1_, *κ*_2_, *κ*_3_ > 0. Note that the investment function increases with respect to the net profit, i.e. profit after wages and debt are paid *z* ≔ 1 − *x*_1_ − *rx*_3_.

It is easy to see that model (4) has a unique equilibrium given by

$$\begin{array}{c} {\bar{x}}_{1}=1-\bar{z}-r{\bar{x}}_{3}, \end{array}$$ (5a)

$$\begin{array}{c} {\bar{x}}_{2}=\Phi^{-1}\left(\alpha \right), \end{array}$$ (5b)

$$\begin{array}{c} {\bar{x}}_{3}=\frac{\nu \left(\alpha +\beta +\lambda \right)-\bar{z}}{\alpha +\beta }, \end{array}$$ (5c)

where $\bar{z}=1-{\bar{x}}_{1}-r{\bar{x}}_{3}$. Moreover, one can show that the equilibrium in (5) is stable as long as ${\bar{x}}_{1}>\nu \lambda$, but might not be finite. This is the main point of the Keen model, which given certain initial conditions, allows for a stable equilibrium with infinite debt, zero wage share, and zero employment rate. Hereafter we denote *R*_*u*_ the set of initial conditions in $R_{+}^{3}$ that lead to this undesired equilibrium, and *R*_*d*_ to its complement, thus related as $R_{+}^{3}=R_{d}\cup R_{u}$.

**Fig 2:**
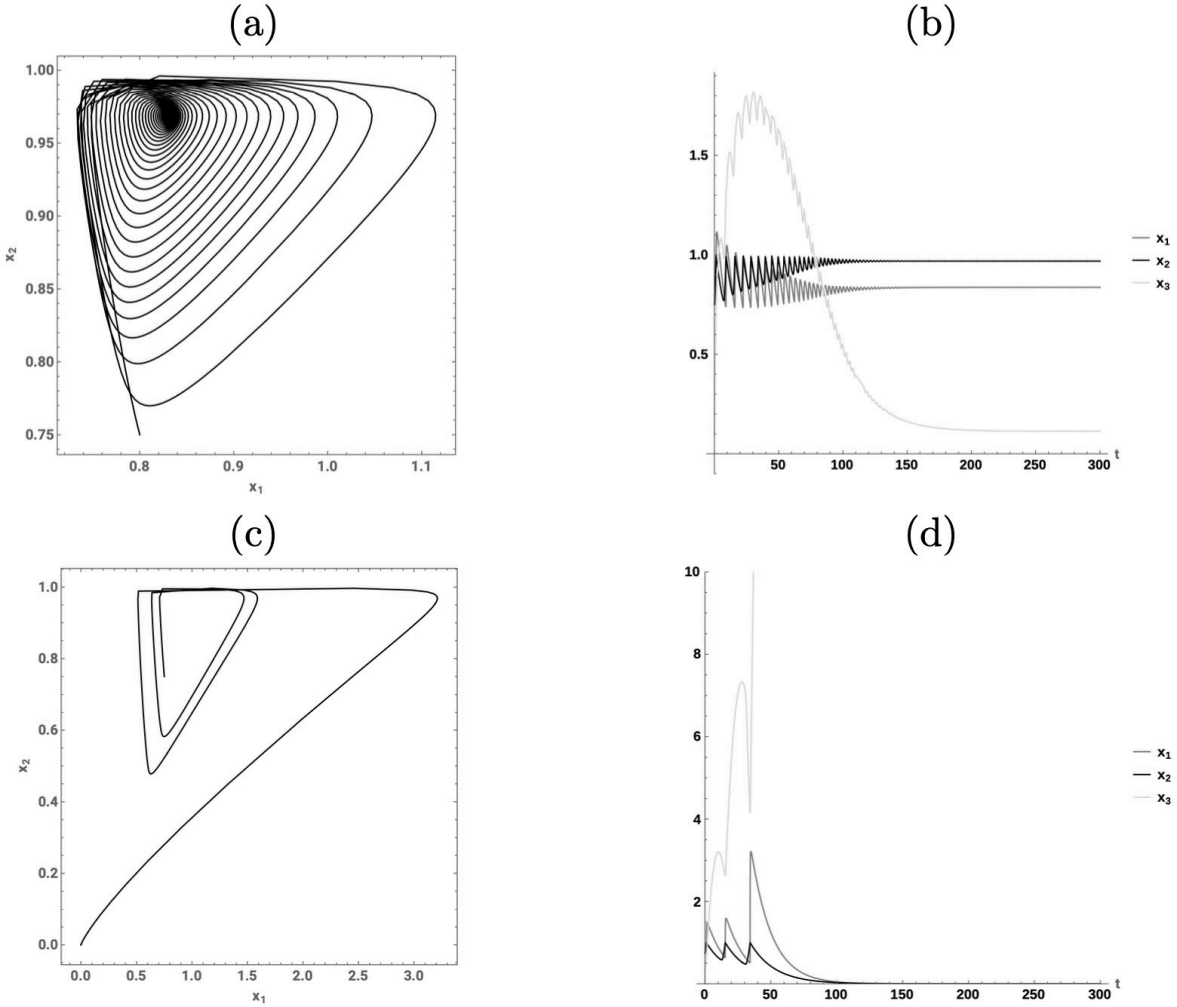
Keen model. Comparison of two Keen models under different initial conditions in the first and second rows of the panel. Subfigs. (a) and (b) portray the convergence to a stable finite equilibrium as the initial condition is given by ***x***_0_ = (0.75, 0.75, 0.1)^⊤^. Subfigs. (c) and (d) show the convergence to the undesired equilibrium, where the debt share grows infinitely, as the initial condition is given by ***x***_0_ = (0.8, 0.75, 0.1)^⊤^.

Subfigs. (a) and (c) in Fig 2 show a realization of the dynamics of the Keen system when the initial condition corresponds to a point in the region *R*_*d*_, and a finite equilibrium is reached. Subfigs. (b) and (d) show realizations when the initial condition belongs to *R*_*u*_. Under the latter setting, the system diverges as wages and employment go to zero while debt increases to infinity.

**Fig 3:**
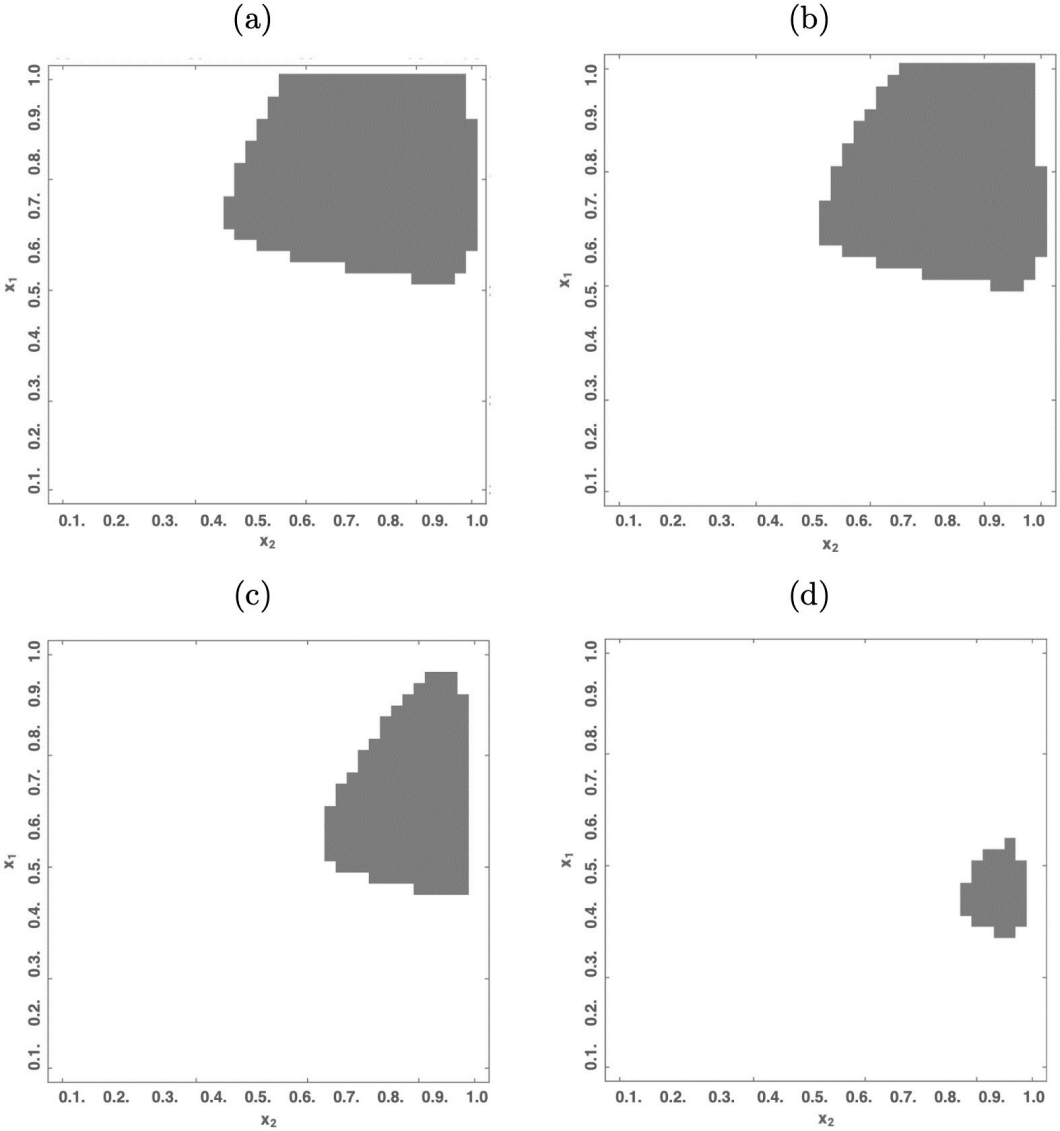
Finite and non-finite equilibria in the Keen model. Each sub-figure shows different combinations of initial conditions for *x*_1_ and *x*_2_, when *x*_3_ is fixed at some level. If the solution of the Keen model a particular combination of initial conditions reaches a desired finite equilibrium, then the cell is colored gray. In any other case, the cell remains white. The fixed levels of *x*_3_ at each sub-figure are: (a) 0.03 (b) 0.1, (c) 2.5 and (d) 5.

Fig 3 shows regions *R*_*d*_ (in gray) and *R*_*u*_ (in white) for different levels of debt share (*x*_3_). Figure (a) portrays the case for *x*_3_ = 0.3, and figures (b), (c), and (d) present the cases for *x*_3_ = 1, *x*_3_ = 2.5 and *x*_3_ = 5 respectively.

Similar to the procedure followed for the Goodwin model, one can select *α*, *β*, and *r* as the inputs to which policymakers can assign values for altering the dynamics of (4). However, the system will not have affine form as in (29), which will be necessary for constructing an appropriate control policy. Therefore, we create a new state *x*_4_ ≔ *r*, and select *u*_1_ ≔ *α*, *u*_2_ ≔ *β* and $u_{3}≔\dot{r}$ as the inputs that policymakers can modify at will to alter the dynamics of the system. The new state satisfies

$$\dot{x_{4}}=\dot{r}=u_{3},$$

which implies that one changes the variation of the interest rate rather *r* itself. This augmentation of (4) has the form of a fourth-dimensional control affine system as in (29). The next proposition shows that the Keen model is accessible.

**Proposition 2.2**. *The augmented Keen model is accessible for all states* ***x*** *in the set* $\left(0,1\right)\times \left(0,1\right)\times R_{+}^{2}$.

*Proof*. Showing accessibility is accomplished by computing (32) and checking that its rank is 4. That is, one can see that

$$\begin{array}{cc} Q(f,g) & =[\begin{array}{ccccccc} f & g_{1} & g_{2} & g3 & \left[f,g_{1}\right] & \left[f,g_{2}\right] & \left[f,g_{3}\right] \end{array}]\\ & =[\begin{array}{ccccccc} x_{1}(\frac{\phi_{1}}{(1-x_{2}{)}^{2}}\;\;-\;\;\phi_{0}) & -x_{1} & 0 & 0 & \frac{2x_{1}x_{2}\phi_{1}}{(1-x_{2}{)}^{3}} & \frac{2x_{1}x_{2}\phi_{1}}{(1-x_{2}{)}^{3}} & 0\\ x_{2}(\Omega \;\;-\;\;\lambda ) & -x_{2} & -x_{2} & 0 & -\frac{\kappa_{1}\kappa_{2}x_{1}x_{2}}{\nu \Lambda^{2}+1} & 0 & \frac{\kappa_{1}\kappa_{2}x_{2}x_{3}}{\nu \Lambda^{2}+1}\\ \Gamma & 0 & 0 & 0 & x_{1}(\frac{\kappa_{1}\kappa_{2}x_{3}}{\nu \Lambda^{2}+1}\;\;-\;\;\frac{\kappa_{1}\kappa_{2}}{\Lambda^{2}+1}\;\;+\;\;1) & 0 & \frac{\kappa_{1}\kappa_{2}x_{3}}{\Lambda^{2}+1}-x_{3}(\frac{\kappa_{1}\kappa_{2}x_{3}}{\nu \Lambda^{2}+1}\;\;+\;\;1)\\ 0 & 0 & 0 & 1 & 0 & 0 & 0 \end{array}], \end{array}$$

where $\Omega =\frac{\kappa_{0}+\kappa_{1}{\text{tan}}^{-1}(\Lambda )}{\nu }$, Λ = *κ*_3_ + *κ*_2_(−*x*_1_ − *x*_3_*x*_4_ + 1), and Γ = *x*_3_(Λ^2^ + 1 − Ω + *x*_4_) + *ν*Ω + *x*_1_ − 1. Here Rank(*Q*(*f*, *g*)) = 4 for $x\in \left(0,1\right)\times \left(0,1\right)\times R_{+}^{2}$, which makes the augmented Keen model accessible in the domain of interest.

For clarity of the exposition, all parameter values used in this manuscript for the Goodwin and the Keen models are listed in Table 1.:

**Table 1:** Keen model parameters.

**Primary Parameters**	**Description**	**Value**
*α*	Rate of growth of labor productivity	0.025
*β*	Rate of growth of labor force	0.02
λ	Capital depreciation constant	0.01
*ν*	Capital-to-output ratio	3
*r*	Real interest rate	0.03
**Nuisance Parameters**	**Usage**	**Value**
*ϕ*_0_	Phillips curve	0.0401042
*ϕ*_1_	Phillips curve	0.000104167
*κ*_0_	Investment function	0.5
*κ*_1_	Investment function	-0.31831
*κ*_2_	Investment function	-63.989
*κ*_3_	Investment function	11.9914

We close this section stressing that the adequacy of both the Goodwin and Keen systems to model economic phenomena have been extensibly explored. We refer the interested reader to [43–47] for the Goodwin model, and to [48, 49] for the Keen model.

## 3 Dynamic control to scape a debt spiral

Our objective in applying control theory to the Keen model is to steer the system from ***x***_0_ ∈ *R*_*u*_ to any point in the stable region ***x***_*τ*_ ∈ *R*_*d*_, as any point in this region will take the system to the desired finite equilibrium. See Table 2 for the exact values of these points.

**Table 2:** Initial conditions.

Variables	*x*_0_	*x*_*τ*_
*x*_1_	0.5	0.87
*x*_2_	0.5	0.87
*x*_3_	0.8	1.40

**Fig 4:**
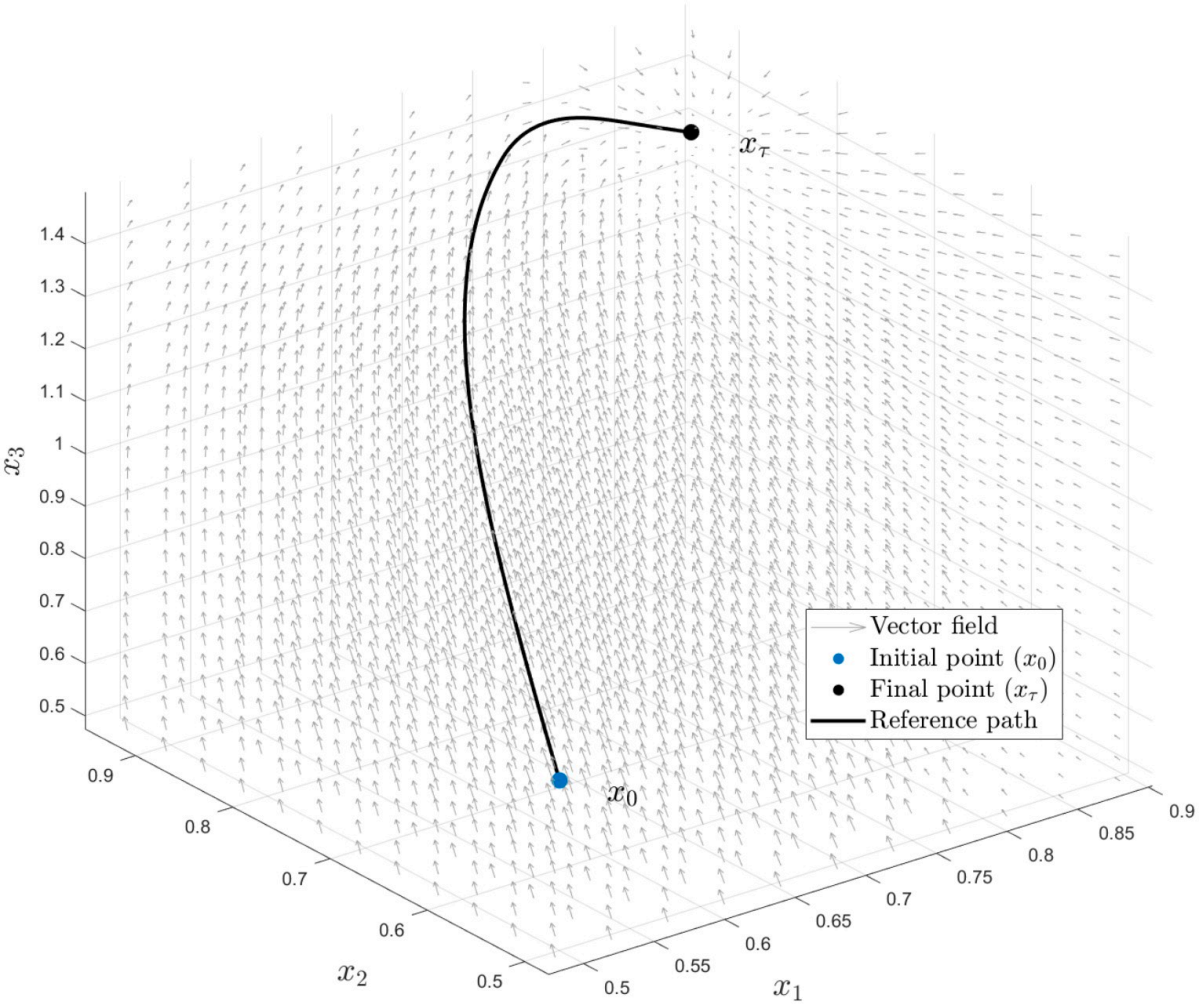
Gradient-based planner. Illustration of the Oracle reference path, generated using Algorithm 1. The path starts at ***x***_0_ = (0.5, 0.5, 0.8)^⊤^ ∈ *R*_*u*_, which is well outside the stable region of the Keen model, and moves towards ***x***_*τ*_ = (0.87, 0.87, 1.4)^⊤^ ∈ *R*_*d*_. The vector field of the Keen model is represented by the gray arrows in the figure to illustrate the dynamics of the system.

This goal can be achieved by tracking some oracle reference path *ℓ* with initial point ***ℓ***(0) = ***x***_0_ and final point ***ℓ***(*τ*) = ***x***_*τ*_ with the aid of a given control mechanism, called control policy. This controlled path can be seen as the solution to a problem that minimizes the gap between the current state of the system and the oracle path at each step in time. Fig 4 illustrates the oracle reference path with its starting and ending points.

### 3.1 Input variables

Input variables or control policies, denoted as vector ***u***, serve as the means of interaction between a policy maker and the economic system described in (4). For the Keen model, we have selected inputs indirectly related to parameters *α*, *β*, and *r*, in the following manner:

$$\begin{array}{c} r=r_{0}+x_{4},\;\;{\dot{x}}_{4}=u_{3}, \end{array}$$ (6)

$$\begin{array}{c} \alpha =\alpha_{0}+x_{5},\;{\dot{x}}_{5}=-\tau_{1}x_{5}+\tau_{1}u_{1}, \end{array}$$ (7)

$$\begin{array}{c} \beta =\beta_{0}+x_{6},\;{\dot{x}}_{6}=-\tau_{2}x_{6}+\tau_{2}u_{2}, \end{array}$$ (8)

where *r*_0_ = 0 and *α*_0_ and *β*_0_ take the values used for the illustration of model (4) in Table 1. In addition, the values *τ*_1_, *τ*_2_ > 0 are set to calibrate their variation latency.

Note that input ***u*** = (*u*_1_, *u*_2_, *u*_3_)^⊤^ interacts with the system trough new states *x*_4_, *x*_5_ and *x*_6_. In the case of *x*_4_ we consider an immediate control over the change in the real interest rate, but for the cases of *α* and *β* we consider a delayed effect as described by (7) and (8) respectively. Hence, system (4) can be re-written isolating the effect of ***u*** over the system as an affine non-linear system given by:

$$\begin{array}{ccc} \dot{x} & = & f(x)+g(x)u,\\ y & = & h(x), \end{array}$$

which can be explicitly written as

$$[\begin{array}{c} {\dot{x}}_{1}\\ {\dot{x}}_{2}\\ {\dot{x}}_{3}\\ {\dot{x}}_{4}\\ {\dot{x}}_{5}\\ {\dot{x}}_{6} \end{array}]=\underset{f(x)}{\underset{︸}{\left[\begin{array}{c} x_{1}[\Phi (x_{2})-\alpha_{0}-x_{5}]\\ x_{2}[\frac{\kappa (x)}{\nu }-\alpha_{0}-x_{5}-\beta_{0}-x_{6}-\lambda ]\\ x_{3}[x_{4}-\frac{\kappa (x)}{\nu }+\lambda ]+[\kappa (x)-(1-x_{1})]\\ 0\\ -\tau_{1}x_{5}\\ -\tau_{2}x_{6} \end{array}\right]}}+\underset{g(x)}{\underset{︸}{\left[\begin{array}{ccc} 0 & 0 & 0\\ 0 & 0 & 0\\ 0 & 0 & 0\\ 0 & 0 & 1\\ \tau_{1} & 0 & 0\\ 0 & \tau_{2} & 0 \end{array}\right]}}[\begin{array}{c} u_{1}\\ u_{2}\\ u_{3} \end{array}],$$ (9a)

$$[\begin{array}{c} y_{1}\\ y_{2}\\ y_{3} \end{array}]=\underset{h(x)}{\underset{︸}{\left[\begin{array}{cccccc} 1 & 0 & 0 & 0 & 0 & 0\\ 0 & 1 & 0 & 0 & 0 & 0\\ 0 & 0 & 1 & 0 & 0 & 0 \end{array}][\begin{array}{c} x_{1}\\ x_{2}\\ x_{3}\\ x_{4}\\ x_{5}\\ x_{6} \end{array}\right]}},$$ (9b)

where *κ*(*x*) has the same meaning as before, but given that *x*_4_ = *r* in (6), we can re-write it as *κ*(*x*) = *κ*(*x*_1_, *x*_3_, *x*_4_) = *κ*_0_ + *κ*_1_tan^−1^(*κ*_2_(1 − *x*_1_ − *x*_4_*x*_3_) + *κ*_3_).

**Fig 5:**
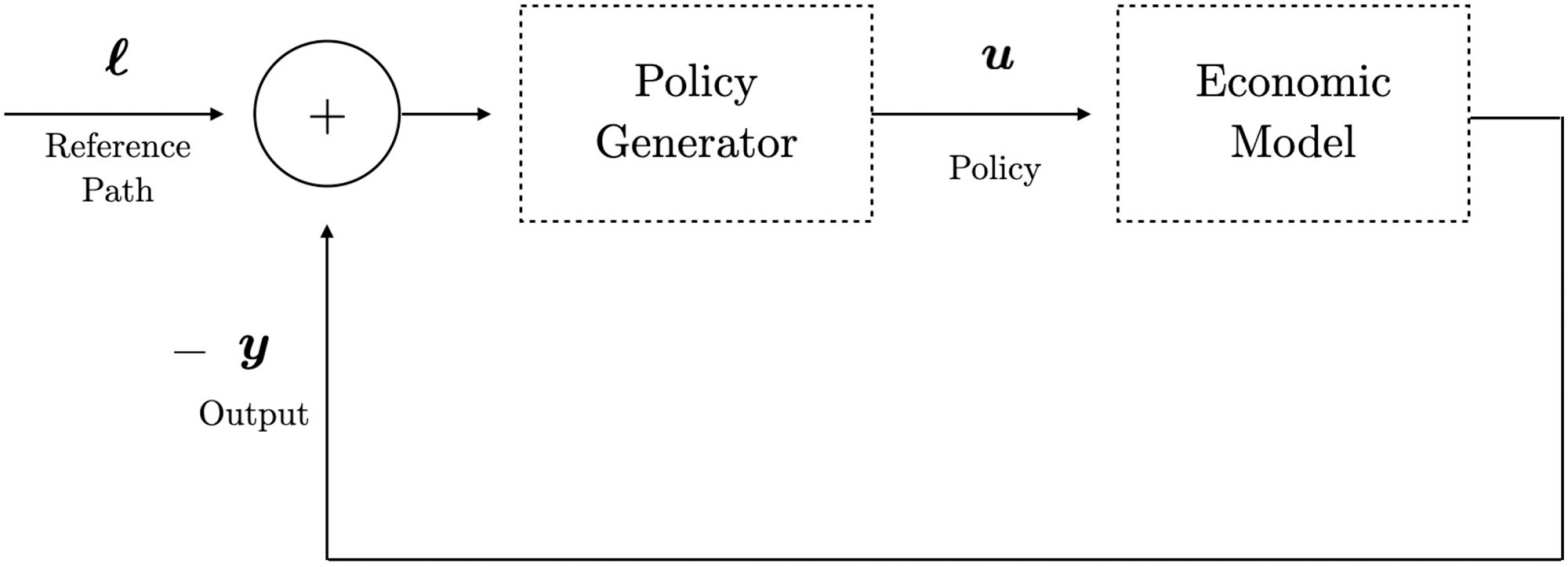
Block diagram representation of a control problem. The policy value ***u*** is the result of some designed policy generator, which takes as input a correction signal given by the difference between reference path ***ℓ*** and output ***y***.

System (9) makes a clear distinction between input variables ***u***, state variables ***x***, and output variables ***y***. For the Keen model, this means that the system can be controlled by modifying inputs ***u*** given by the growth rates of labor productivity (*x*_5_), labor force (*x*_6_), and interest rates (*x*_4_). The impact on these quantities is then carried into the system’s full state ***x***, out of which only certain output quantities ***y***, given by the state variables in the Keen model (4), are monitored. See Fig 5 for a general illustration of the control process.

### 3.2 Exact linearization

To control the inputs in model (9) it is convenient to first linearize it. Here we employ a technique called *feedback linearization*, which allows for an exact linearization of the system, in contrast to e.g. Taylor linearization, which is only an approximation. The main idea of the method is to find a feedback linearizing control law (see Eq (42) in subsection B.2 of the appendix) that linearizes an affine nonlinear system exactly. The following theorem shows that for the particular case of system (9) this control law exists.

**Theorem 3.1**. *There exists a unique feedback linearizing control law* ***u***_*K*_(***x***) *for system* (9) *given by*

$$\begin{array}{c} u_{K}\left(x\right)=D_{K}{\left(x\right)}^{-1}\left(-\Theta_{K}\left(x\right)+v\right), \end{array}$$ (10)

*where*

$$\begin{array}{cc} D_{K}\left(x\right) & =[\begin{array}{ccc} -x_{1} & -x_{2} & 0\\ 0 & -x_{2} & 0\\ 0 & L_{g_{2}}f_{3} & L_{g_{3}}f_{3} \end{array}], \end{array}$$ (11)

$L_{g_{2}}f_{3}=-\frac{6.8x_{2}x_{3}}{{\left(64x_{1}+64x_{3}x_{4}-52\right)}^{2}+1}$ *and* $L_{g_{3}}f_{3}=\frac{6.8x_{3}^{2}-20x_{3}}{{\left(64x_{1}+64x_{3}x_{4}-52\right)}^{2}+1}+x_{3}$, *in the domain x*_1_ ∈ (0, 1), *x*_2_ ∈ (0, 1), $x_{3}\in R$ *and*

$$\begin{array}{c} x_{4}\neq 0.003125x_{3}^{-2}\left(260x_{3}-320x_{1}x_{3}\pm 2.23607{\left(95x_{3}^{2}-34x_{3}^{3}\right)}^{1/2}\right). \end{array}$$ (12)

*In addition*, ***v*** *and* Θ_*K*_(***x***) *are three dimensional vectors, where the first is a free vector and the second has elements given by*

$$\begin{array}{c} \Theta_{1}\left(x\right)=\Theta_{11}\left(x\right)+\Theta_{12}\left(x\right)\Theta_{13}\left(x\right), \end{array}$$ (13a)

$$\begin{array}{c} \Theta_{2}\left(x\right)=\Theta_{21}\left(x\right)+\Theta_{22}\left(x\right)+\left(\Theta_{23}\left(x\right)+\Theta_{24}\left(x\right)\right)/\Theta_{25}\left(x\right)+\Theta_{26}\left(x\right), \end{array}$$ (13b)

$$\begin{array}{cc} \Theta_{3}\left(x\right) & =\left(\Theta_{31}\left(x\right)+\Theta_{32}\left(x\right)\right)\left(\Theta_{33}\left(x\right)+\Theta_{34}\left(x\right)+\Theta_{35}\left(x\right)\right)+\\ & \;\;\left(\Theta_{36}\left(x\right)+\Theta_{37}\left(x\right)\right)\Theta_{38}\left(x\right), \end{array}$$ (13c)

*where we have omitted subscript K for each component to ease the notation. More specifically*

$$\begin{array}{ccc} \Theta_{11}\left(x\right) & = & 10x_{1}x_{5}+x_{1}(x_{5}-\frac{10^{-4}}{{\left(x_{2}-1\right)}^{2}}+0.065{)}^{2},\\ \Theta_{12}\left(x\right) & = & (2.1\times 10^{-4}x_{1}x_{2}\left(x_{5}+x_{6}+0.11\;{\text{tan}}^{-1}\left(64x_{1}+64x_{3}x_{4}-52\right)-0.11\right)),\\ \Theta_{13}\left(x\right) & = & {\left(x_{2}-1\right)}^{-3},\\ \Theta_{21}\left(x\right) & = & x_{2}{\left(x_{5}+x_{6}+0.11\;{\text{tan}}^{-1}\left(64x_{1}+64x_{3}x_{4}-52\right)-0.11\right)}^{2},\\ \Theta_{22}\left(x\right) & = & 10x_{2}x_{5}+10x_{2}x_{6},\\ \Theta_{23}\left(x\right) & = & 6.8x_{2}x_{4}\left(x_{1}-0.32\;{\text{tan}}^{-1}\left(64x_{1}+64x_{3}x_{4}-52\right)\right),\\ \Theta_{24}\left(x\right) & = & 6.8x_{2}x_{4}\left(x_{3}\left(x_{4}+0.11\;{\text{tan}}^{-1}\left(64x_{1}+64x_{3}x_{4}-52\right)-0.16\right)-0.5\right),\\ \Theta_{25}\left(x\right) & = & {\left(64x_{1}+64x_{3}x_{4}-52\right)}^{2}+1,\\ \Theta_{26}\left(x\right) & = & \left(6.8x_{1}x_{2}\left(x_{5}-10^{-4}{\left(x_{2}-1\right)}^{-2}+0.065\right)\right){\left({\left(64x_{1}+64x_{3}x_{4}-52\right)}^{2}+1\right)}^{-1},\\ \Theta_{31}\left(x\right) & = & x_{1}-0.32\;{\text{tan}}^{-1}\left(64x_{1}+64x_{3}x_{4}-52\right),\\ \Theta_{32}\left(x\right) & = & x_{3}\left(x_{4}+0.11\;{\text{tan}}^{-1}\left(64x_{1}+64x_{3}x_{4}-52\right)-0.16\right)-0.5,\\ \Theta_{33}\left(x\right) & = & x_{4}+0.11\;{\text{tan}}^{-1}\left(64x_{1}+64x_{3}x_{4}-52\right),\\ \Theta_{34}\left(x\right) & = & -\left(20x_{4}\right){\left({\left(64x_{1}+64x_{3}x_{4}-52\right)}^{2}+1.0\right)}^{-1},\\ \Theta_{35}\left(x\right) & = & \left(6.8x_{3}x_{4}\right){\left({\left(64x_{1}+64x_{3}x_{4}-52\right)}^{2}+1.0\right)}^{-1}-0.16,\\ \Theta_{36}\left(x\right) & = & -x_{1}\left(6.8x_{3}\right){\left({\left(64x_{1}+64x_{3}x_{4}-52\right)}^{2}+1\right)}^{-1},\\ \Theta_{37}\left(x\right) & = & 20x_{1}{\left({\left(64x_{1}+64x_{3}x_{4}-52\right)}^{2}+1\right)}^{-1}+1),\\ \Theta_{38}\left(x\right) & = & x_{5}-10^{-4}{\left(x_{2}-1\right)}^{-2}+0.065. \end{array}$$

*Proof*. The proof follows the construction of (42) as provided in subsection B.2 of the Appendix. The computations of ***D***_*K*_(***x***) and Θ_*K*_(***x***) follow from (39) and (41) respectively. To make (9) exactly linearizable one must show that ***D***_*K*_(***x***) is invertible in the domain *x*_1_ ∈ (0, 1), *x*_2_ ∈ (0, 1), $x_{3}\in R$ and $x_{4}\in R$ such that (12) holds, which can be trivially achieved given that *x*_4_ is controlled by the modeler via *u*_3_.

Replacing the results in theorem 3.1 in (40), we obtain the feedback linearized version of system (9) in Byrnes-Isidori form as:

$$\begin{array}{c} \dot{z}=Mz+Nv, \end{array}$$ (14a)

$$\begin{array}{c} y=Wz, \end{array}$$ (14b)

where $z\in R^{6}$, $v,y\in R^{3}$, and matrices ***M***, ***N*** and ***W*** are defined as

$$\begin{array}{c} M=[\begin{array}{cccccc} 0 & 1 & 0 & 0 & 0 & 0\\ 0 & 0 & 0 & 0 & 0 & 0\\ 0 & 0 & 0 & 1 & 0 & 0\\ 0 & 0 & 0 & 0 & 0 & 0\\ 0 & 0 & 0 & 0 & 0 & 1\\ 0 & 0 & 0 & 0 & 0 & 0 \end{array}],N=[\begin{array}{ccc} 0 & 0 & 0\\ 1 & 0 & 0\\ 0 & 0 & 0\\ 0 & 1 & 0\\ 0 & 0 & 0\\ 0 & 0 & 1 \end{array}],W=[\begin{array}{cccccc} 1 & 0 & 0 & 0 & 0 & 0\\ 0 & 0 & 1 & 0 & 0 & 0\\ 0 & 0 & 0 & 0 & 1 & 0 \end{array}]. \end{array}$$ (15)

**Fig 6:**
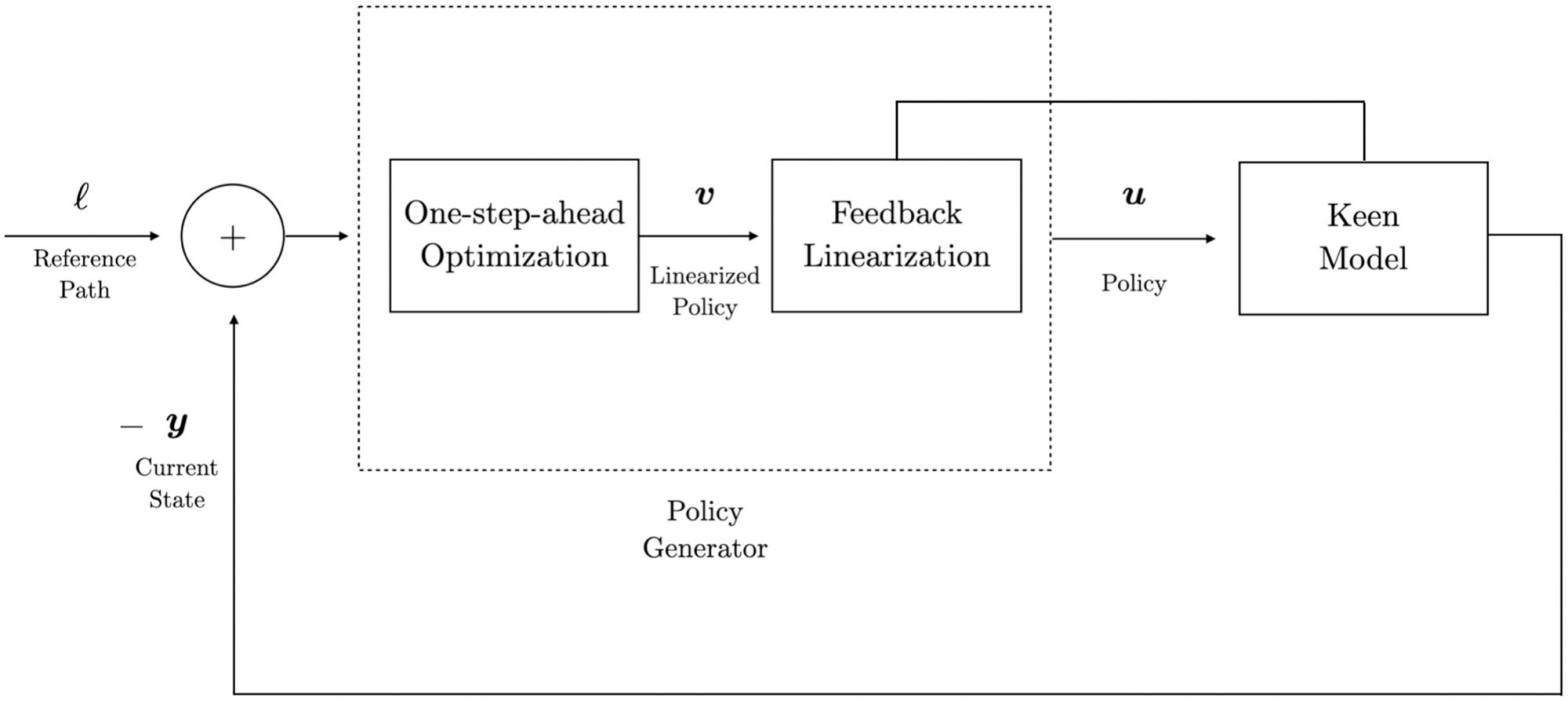
Block diagram representation of the control problem for the Keen model. The diagram includes the specific components of the policy generator articulated via the designed control policy ***u***: one-step-optimal-control and feedback linearization. The rest of the diagram is exactly as described in Fig 5.

System (14) is the version of the Keen model that is utilized hereafter to apply standard linear control techniques through changes over input ***v***. See Fig 6 for a detailed scheme of the control process.

### 3.3 Controlling the Keen model

Consider an Euler discretization of (14) for some fixed sampling time *δ* as

$$\begin{array}{c} z[k+1]=(I+\delta M)z[k]+Nv[k], \end{array}$$ (16a)

$$\begin{array}{c} y[k]=Wz[k] \end{array}$$ (16b)

where ***M***, ***N***, and ***W*** where defined in (15), and δ∈R represents some sampling time. Given certain reference signal ***ℓ***[*k*], one can define a quadratic loss function for error ***e***[*k*] = ***y***[*k*] − ***ℓ***[*k*] as

$$\begin{array}{c} J=e{\left[k\right]}^{⊤}Qe\left[k\right], \end{array}$$ (17)

where ***Q*** is a weight matrix affecting the coordinates of the error. Note that function (17) can be expressed explicitly in terms of the control policy ***u***[*k*] by replacing vector ***v***[*k*] from (10) into the definition of ***y***[*k*] in (16). Thus, we can re-write it as

$$\begin{array}{c} J\left(u\left[k\right]\right)=e{\left(u\left[k\right]\right)}^{⊤}Qe\left(u\left[k\right]\right), \end{array}$$ (18)

$$\begin{array}{c} e(u[k])=W(Mz[k]+N(D(z[k])u[k]+\Theta (z[k])))-\ell [k], \end{array}$$ (19)

where the loss function is conveniently expressed in terms of the control policy vector. In addition, to bound the search space of the policy vector, we consider the following constraints: i) |*u*_*i*_| ≤ *B*_*u*_, *i* = 1, 2, 3 where *B*_*u*_ is some real scalar; and ii) *r*_min_ < *r* < *r*_max_, which can be written in terms of *u*_*i*_ as (*r*_min_ − *x*_4_)/*δ* ≤ *u*_3_ ≤ (*r*_max_ − *x*_4_)/*δ*, where *δ*. With the latter considerations, we can express our final optimization problem as

$$\begin{array}{c} u^{*}\left[k\right]=\text{arg}\;\underset{u[k]}{\text{min}}\{J\left(u\left[k\right]\right):|u_{1}|\leq B_{u},\left|u_{2}\right|\leq B_{u},\;(\frac{r_{\text{min}}-x_{4}}{\delta })\leq u_{3}\leq (\frac{r_{\text{max}}-x_{4}}{\delta })\}, \end{array}$$ (20)

which is convex in ***u***[*k*], and thus existence and uniqueness of the solution are guaranteed.

## 4 Simulation results

This section presents a simulation study to showcase the methodology presented in the previous section. This synthetic experiment utilizes the parameters specified in Table 2, following [36], thus the use of real data remains open for future research. We begin by providing an algorithm to generate a reasonable tracking trajectory for the Keen model (4); continue presenting two simulation scenarios, and close by inspecting some numerical results.

### 4.1 Reference path

To obtain an appropriate reference path, a gradient-based planner (GBP) algorithm is introduced, which explicitly embeds Keen’s model dynamical equations in the path generation between two pre-established locations in the state space. Let ***x***_0_ ∈ *R*_*u*_ and ***x***_*τ*_ ∈ *R*_*d*_, where *R*_*d*_ and *R*_*u*_ denote desired and undesired regions in the state-space respectively.

Let *F*_*x*_ denote the vector field of (4) and *F*_*K*_ denote an attractive potential field with attractive constant *K* centered at ***x***_0_, that strengthens with the proximity to ***x***_*τ*_. Define the reference path as the minimum resistance path from ***x***_0_ to ***x***_*τ*_ under the force field generated by *F*_*s*_ = *F*_*K*_ + *F*_*x*_. The construction of such reference path can be recursively performed as presented in Algorithm (1).

**Algorithm 1** Gradient Based Planner

1: **procedure** GBP(***x***_0_, ***x***_*τ*_, *F*_*K*_, *F*_*x*_, *τ*, *δ*)    ▹ Path between ***x***_0_ and ***x***_*τ*_

2:  *F*_*s*_ ← *F*_*K*_ + *F*_*x*_

3:  **while** *i* ≠ *τ* ∨ ||***x***_*τ*_ − *loc*|| < *δ* **do**

4:   *loc* ← ***x***_0_

5:   Δ ← *interpolate*(*F*_*s*_, *loc*)

6:   *loc* ← *loc* + *δ*/(*τ*‖*δ*‖)

7:   *route* ← *stack*(*route*, *loc*)

8:   *i* ← *i* + 1

9:  **return** *route*

It is worth noting that there is a trade-off in the construction process of the reference path. Namely, while a shorter path might be desirable, it does require more control effort. This trade-off should can be managed by the selection of *K*, in a way that the path is short but the effort is feasible. Another important factor is the singularity of the system at *x*_2_ = 1 due to the Phillips curve. If the attractive force is not strong enough, the path generated by *F*_*s*_ could enter this singularity and never leave. In this study, the value of *K* was selected balancing control effort and length of the reference path.

### 4.2 Simulation scenarios

The control policies enter the system in the form of *u*_1_, *u*_2_, and *u*_3_, representing changes in the rates of growth of labor productivity (*α*), labor force (*β*) and interest rates (*r*) respectively, starting from a certain initial state of the economy ***x***_0_ ∈ *R*_*u*_. From this point onwards the control policies presented in section 3 are utilized to transfer the economy from ***x***_0_ towards *R*_*d*_. We selected the specific point ***x***_0_ = (0.5, 0.5, 0.8)^⊤^ considering that, while in the undesired region, it remains close to the frontier with the desired region, which is easy to note by inspecting Fig 3, where the cases for *x*_3_ = 0.1 and *x*_3_ = 5 are represented in panels (b) and (c) respectively.

The reference path in Fig 4 is generated utilizing Algorithm 1 in Subsection 4.1, so that control can be applied to track it. The minimization procedure described in Subsection 3.3 minimizes the error between output and a reference path using the following parameter values: *B*_*u*_ = 1, *r*_*min*_ = 0, *r*_*max*_ = 0.5. Furthermore, we considered two main illustrative scenarios. In the first case, we presumed low latency (*τ*_1_ = *τ*_2_ = 1000) in any of the controlled variables, and in the second case, we assume high latency (*τ*_1_ = *τ*_2_ = 1), with the exception of interest rates. We stress that the second case is more realistic as control policies ***u***_1_ and ***u***_2_ are not expected to be executed with immediacy, and include the first scenario for comparison purposes exclusively.

### 4.3 Results

**Fig 7:**
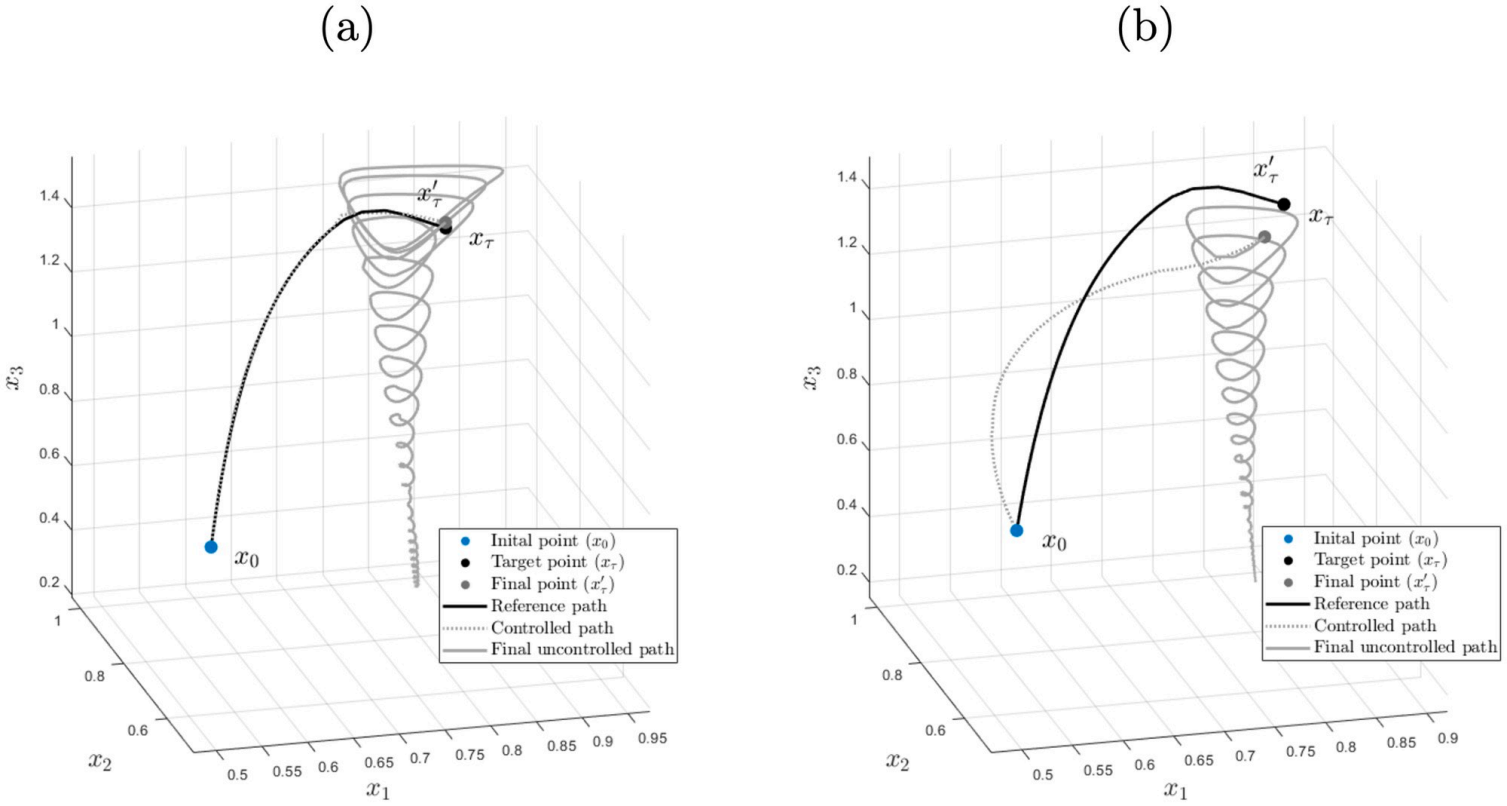
Control of Keen’s model under two latency scenarios. Scenario (a) presumes low latency (*τ*_1_ = *τ*_2_ = 1000), while scenario (b) considers high latency (*τ*_1_ = *τ*_2_ = 1) in the control policies. As can be seen, in scenario (a), the target point (black) is almost on top of the final point (gray). In fact, the reference path (continuous black line) and the controlled path (gray dashed line) are very close to one another, indicating that the system can be controlled with ease. In scenario (b) we observe that the final (black) and target (gray) points are further from each other and that the controlled path does not follow the reference path with the same proximity as in the previous scenario, which resulted in a slower response to the applied control policies due to the added latency. Nonetheless, in both scenarios, we see that the Keen model is effectively controlled from an initial point (*x*_0_) towards a small enough neighborhood of final destination (*x*_*τ*′_) within the stable region of the Keen system. Once the control policy is released in such neighborhood the uncontrolled path follows a stable downward (continuous gray line).

We illustrate the control process in Fig 7, where the control starts at ***x***_0_ = (0.5, 0.5, 0.8)^⊤^ and finishes in the vicinity of ***x***_*τ*_ = (0.87, 0.87, 1.4)^⊤^ ∈ *R*_*d*_. The same figure shows the vector field of the Keen model as the collection of gray arrows, portraying the dynamics of the system. As expected, when low latency is presumed, the tracking of the reference path is performed with barely any misalignments, as shown in panel (a) of Fig 7. However, if high latency is considered, as it is a more realistic scenario, tracking of the reference path is slow and achieved with a certain difficulty. In fact, Fig 7 differentiates the final point ($x_{\tau }^{\prime }$) from the target point (***x***_*τ*_) to account for the fact that the target might only be reached within a certain neighborhood, which is good enough for our purposes as long as $x_{\tau }^{\prime }\in R_{d}$, which is indeed the case.

**Fig 8:**
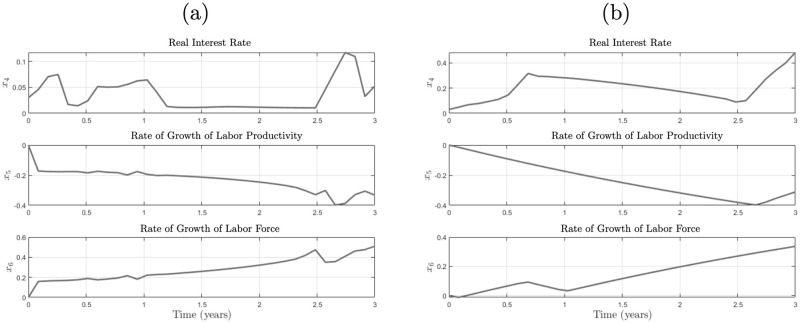
Control of Keen’s model under different latency scenarios. (a) shows the tracking of trajectories *x*_4_, *x*_5_ and *x*_6_ with low latency (*τ*_1_ = *τ*_2_ = 1000), and (b) shows the same for the case of high latency (*τ*_1_ = *τ*_2_ = 1). As can be seen, in the low latency case, the control policies are able to fluctuate more freely than in the high latency case, as the effect of the latter is the reduction in the velocity of the policies. In both cases, however, is noticeable the reduction in the rate of growth in labor productivity, as well as the increment in the rate of growth of the labor force, while maintaining positive values for the interest rates, as enforced by the conditions in equation (20).

Fig 8 shows the trajectories of the resulting levels of *α*, *β*, and *r* for the two latency cases in panels (a) and (b). The main difference between the two cases can be seen in the behavior of interest rates. While in the heavy latency case, the goal can only be achieved by increasing interest rates to levels surpassing 0.4, in the low latency case, interest rates are modified within a narrower radius. Besides the increment of interest rates, the recipe for leaving a debt spiral seems to pass through decreasing the rate of growth of labor productivity and increasing the rate of growth of the labor force, which might seem counter-intuitive at first sight. However, since Keen’s model inherits Goodwin’s trade-off behavior between firms and households, the real meaning of this behavior is that either production must be reduced, and more of the utility should go to households or more labor should be hired.

We stress that the success of the former control strategy assumes that the Keen model provides a complete characterization of the economy, and it is conditioned to the availability of control policies in interest rates (***u***_3_) and the growth rate of labor productivity (via ***u***_1_), and the growth rate of the labor force (via ***u***_2_). Lastly, for the former recipes to be successful, they must be deployed exactly as described, starting from point ***x***_0_ = (0.5, 0.5, 0.8)^⊤^, under the aforementioned latency conditions.

## 5 Conclusions

This manuscript shows that the Keen model can be controlled to steer the economy from an initial undesirable location, where debt grows very fast, to a desired location where all states converge over time to a finite, and well-balanced equilibrium. To accomplish this goal, the model was dynamically extended and feedback linearized so that one-step-ahead optimal control can be applied. It is thus argued that the linearized version of the Keen model can be used as a baseline reference to obtain policies that control debt share within an economy in critical situations. We refer to our findings as a baseline reference as the success of the suggested policies depends crucially on the mild controllability of the productivity rate of growth, and the labor force rate of growth, which are assumed.

It is worthwhile noting that under the Keen model, a financial crisis is not considered an anomaly, or a black swan, but a cyclical non-periodic phenomenon, that will, unequivocally, repeat itself. In this sense we consider our results to be of great significance for high-level policy-making since they allow for a deeper understanding of the financial system and the control policies that can be utilized to navigate the next financial crisis. In particular, theorem 3.1 shows that there exists a unique control law for the Keen model, which can be used to exit a crisis under certain specific conditions.

This work can be extended in various directions. From a statistics perspective it is worth noting that the empirical testing of the Keen model has not been as vastly evaluated as in the case of the Goodwin model, and systematic validation of the Keen model is foundational for any future developments. From a more engineering perspective, the reference generation algorithm can be improved by considering control action and effort as it is done in path planning. Furthermore, control effort can also be included in the cost function of the minimization problem for the policy generation, so that input variability can also be controlled. Lastly, the stochastic and data-driven extensions are currently under investigation and are deferred to future publications.

## 6 Appendix

This appendix presents the basic ideas of linear control systems and feedback linearization that are required to follow the steps in section 3. The contents are based on [21, 38, 39], and include linear and geometric-based techniques.

In general, a *control system* with states $x\in R^{n}$ and inputs $u\in R^{m}$ is any system that has the form

$$\dot{x}=f(x,u)$$ (21)

$$x(0)=x_{0}.$$ (22)

In addition, the system is called *controllable* if for any given $x_{0},x_{\tau }\in R^{n}$ and τ∈R, there exists an input $u:R\to R^{m}$ such that the problem

$$\begin{array}{c} \dot{x}=f\left(x,u\right) \end{array}$$ (23)

$$\begin{array}{c} x\left(0\right)=x_{0} \end{array}$$ (24)

$$\begin{array}{c} x\left(\tau \right)=x_{\tau } \end{array}$$ (25)

has a solution.

## A Control systems and their controllability

A control system is called *linear control system* if it can be written as

$$\begin{array}{c} \dot{x}=\;Ax+Bu, \end{array}$$ (26a)

$$\begin{array}{c} y=\;Cx, \end{array}$$ (26b)

where $x\in R^{n}$, $u\in R^{m}$, $y\in R^{q}$ and ***A***, ***B*** and ***C*** are matrices of appropriate dimensions. Additionally, we say that the system is controllable if the rank of its *controllability matrix*

$$\begin{array}{c} C_{A,B}=\left[B\;AB\;\cdots \;A^{n-1}B\right] \end{array}$$ (27)

equals *n*. This implies that *n* directional vectors can be employed to steer ***x*** to any desired place in $R^{n}$. A control policy ***u*** = ***Kx***, $K\in R^{m\times n}$ is known as *state feedback*, and it provides the desired policy by selecting the components of ***K*** appropriately. If such ***K*** exists, the system (26) turns into

$$\begin{array}{c} \dot{x}=\left(A+BK\right)x, \end{array}$$ (28a)

$$\begin{array}{c} y=Cx, \end{array}$$ (28b)

and its trivial solution is ***x*** = ***C*** exp{(***A*** + ***BK***)*t*}***x***_0_, which can be made zero by choosing ***K*** in a way that the eigenvalues of ***A*** + ***BK*** are negative and large enough. Hence, the system is guaranteed to reach the origin after some time commanded by how fast the exponential decays, which is a tuning parameter for the control policy.

When a control system is not linear, it is still possible to Taylor linearize it so that one can employ techniques developed for linear systems, however, such Taylor linearization is only valid over a small neighborhood of an operating point. This limits the applicability of the control policy, the quantification of uncertainty is not accurate, and no guarantees of performance can be claimed.

In a more general setting, hereafter we consider the class of affine nonlinear systems. That is, systems of the form

$$\begin{array}{c} \dot{x}=\;f\left(x\right)+g\left(x\right)\;u,\;x\left(0\right)=x_{0}, \end{array}$$ (29a)

$$\begin{array}{c} y=\;h(x) \end{array}$$ (29b)

with $x\in R^{n}$ and *f*, *g* and *h* sufficiently smooth in a domain of $R\subset R^{n}$.

### A.1 Controllability of affine nonlinear systems

Bringing in concepts from differential geometry [50], the set of all smooth vector fields on a state space is denoted by X(R). This forms a real vector field and a Lie algebra under the multiplication defined by the bracket operation

[·,·]:X(R)×X(R)→X(R) (30)

$$:\;f,g\mapsto \left[f,g\right]\left(x\right)=\frac{\partial g}{\partial x}f\left(x\right)-\frac{\partial f}{\partial x}g\left(x\right).$$ (31)

For each constant input, a vector field is defined by (21), and the collection of such vector fields is denoted by $V^{0}\left(R\right)$. The smallest subalgebra of X(R) containing $V^{0}\left(R\right)$ is denoted by V(R). Furthermore, denote by $TV{\left(R\right)}_{x}$ the space of tangent vectors spanned by the vector fields in V(R) at *x*. A system is said to satisfy the *controllability rank condition at x_0_* if the dimension of $TV{\left(R\right)}_{x_{0}}$ is *n*.

However, not all vector fields are relevant to construct $TV{\left(R\right)}_{x}$. Here it will be enough to focus on vector fields built iteratively by brackets of the form

$$\begin{array}{c} ad_{f}^{k}g\left(x\right)=\left[f,ad_{f}^{k-1}g\left(x\right)\right], \end{array}$$

where $ad_{f}^{0}=f$. Thus, for an affine system of the form $\dot{x}=f\left(x\right)+\sum_{i=1}^{m}g_{i}\left(x\right)u_{i}$ the controllability rank condition at *x* reduces to checking the rank of the matrix

$$\begin{array}{c} Q\left(f,g\right)=[f\left(x\right)\;g_{1}\left(x\right)\;\cdots \;g_{m}\left(x\right)\;ad_{f}^{1}g_{1}\left(x\right)\;\cdots \;ad_{f}^{1}g_{m}\left(x\right)\cdots ad_{f}^{r}g_{m}\left(x\right)] \end{array}$$ (32)

equals *n* for some *m* > 0. When this condition is satisfied, then such a system is called *accessible*. The property of accessibility simply means that the set of states that can be reached from a state *x* through some admissible input is not empty. Therefore, stronger notions of controllability exist for nonlinear systems, but these are outside the scope of this manuscript. More details and stronger notions of controllability for nonlinear systems can be found in [51–54]. Nevertheless, one can see that (32) reduces to (27) for linear systems as in (26) due to the Cayley-Hamilton Theorem [39].

In the following section, we present a technique that guarantees the manipulation of systems of the form (29) avoiding Taylor linearization, called *feedback linearization*. This method allows for the design of a matrix ***K***, as in (28), for tracking a desired reference path without resorting to approximations.

## B Feedback linearization

Feedback linearization is a technique used to obtain a linear system that exactly represents the dynamics of an *affine system* as presented in (29). To accomplish this goal, a nonlinear change of variables is needed. The procedure to exactly linearize an affine system is described next. Consider first the definition of a Lie derivative.

**Definition 7.1**. *Let f and h be smooth functions. Denote by*

$$\begin{array}{c} L_{f}h\left(x\right)=\frac{\partial h}{\partial x}f\left(x\right) \end{array}$$

*the vector field* Lie derivative *of h with respect to f, and*

$$\begin{array}{c} L_{f}^{k}h\left(x\right)=L_{f}\left(L_{f}^{k-1}h\left(x\right)\right)=\frac{\partial L_{f}^{k-1}h\left(x\right)}{\partial x}f\left(x\right) \end{array}$$

*is the k-th iterated Lie derivative of h with respect to f*.

The relative degree of an affine nonlinear system is then provided by the next definition.

**Definition B.2**. *System* (29) *is said to have* relative degree 0 ≤ *p* ≤ *n* *in the region* $R_{x_{0}}\in R^{n}$ *if for all* $x\in R_{x_{0}}$

$$\begin{array}{c} L_{g}L_{f}^{i-1}h\left(x\right)=0\;for\;i=1,2,\ldots ,p-1, \end{array}$$

$$\begin{array}{c} L_{g}L_{f}^{p-1}h\left(x\right)\neq 0, \end{array}$$

### B.1 SISO systems

For a system with a single input and a single output (SISO), the *k*-th derivative of y∈R in (29) for a neighborhood of *t*_0_ = ***x***(0) can be written as

$$\begin{array}{c} y^{\left(p\right)}\left(t_{0}\right)=L_{f}^{p}h\left(x_{0}\right)+L_{g}L_{f}^{p-1}h\left(x_{0}\right)u\left(t_{0}\right), \end{array}$$ (33)

and for relative degree *p* > *k* it holds that $y^{\left(k\right)}=L_{f}^{k}h\left(x\right)$. Therefore, the relative degree *p* of a system is equal to the number of times the output must be differentiated to have the input appear explicitly. The functions $h\left(x\right),L_{f}h\left(x\right),\ldots ,L_{f}^{p-1}h\left(x\right)$ define a key local coordinate transformation for the system about ***x***_0_ and thus define a new set of coordinates ***z*** = *T*(***x***) where $T:R^{n}\to R^{p}$, explicitly given by

$$\begin{array}{c} T\left(x\right)=[\begin{array}{c} h(x)\\ L_{f}h\left(x\right)\\ \vdots \\ L_{f}^{p-1}h\left(x\right) \end{array}]. \end{array}$$ (34)

After this change of variables, the resulting system is:

$$\dot{h}=z_{1}$$ (35a)

$${\dot{z}}_{1}=z_{2}$$ (35b)

⋮ (35c)

$${\dot{z}}_{p-2}=z_{p-1}$$ (35d)

$${\dot{z}}_{p-1}=L_{f}^{p}h\left(x_{0}\right)+L_{g}L_{f}^{p-1}h\left(x_{0}\right)u\left(t_{0}\right).$$ (35e)

We say that an affine system is *exactly linearizable* if there exists an invertible $D≔L_{g}L_{f}^{p-1}h\left(x_{0}\right)$. If this is the case, one can define

$$\begin{array}{c} u=D^{-1}\left(v-L_{f}^{p}h\left(x_{0}\right)\right) \end{array}$$ (36)

and the linear system (35) can be re-written as:

$$\begin{array}{cc} \left[\begin{array}{c} \dot{h}\\ {\dot{z}}_{1}\\ \;\vdots \\ {\dot{z}}_{p-2}\\ {\dot{z}}_{p-1} \end{array}\right] & =[\begin{array}{cccccc} 0 & 1 & 0 & \cdots & 0 & 0\\ 0 & 0 & 1 & \cdots & 0 & 0\\ \vdots & \vdots & \vdots & \ddots & \vdots \\ 0 & 0 & 0 & \cdots & 0 & 1\\ 0 & 0 & 0 & \cdots & 0 & 0 \end{array}][\begin{array}{c} h\\ z_{1}\\ \;\vdots \\ z_{p-2}\\ z_{p-1} \end{array}]+[\begin{array}{c} 0\\ 0\\ \;\vdots \\ 0\\ 1 \end{array}]u. \end{array}$$ (37a)

In this case, the system has the form (26), and its controllability matrix is given by

$$\begin{array}{c} C_{A,B}=[\begin{array}{ccccc} 0 & 0 & \cdots & 0 & 1\\ 0 & 0 & \cdots & 1 & 0\\ \vdots & \;\vdots & \cdots & \vdots & \vdots \\ 0 & 1 & \cdots & 0 & 0\\ 1 & 0 & \cdots & 0 & 0 \end{array}] \end{array}$$ (38)

which has full rank and thus the system is controllable. We highlight the generality of this result, which means that if the original system is exactly linearizable (***D*** is invertible), then the resulting linear system is always controllable.

### B.2 MIMO systems

For a system with *ℓ* outputs and *m* inputs, one can compute the relative degree for each output, say *p*_*i*_ for *i* = 1, …, *ℓ*, by simply computing derivatives until any of the inputs appear. This defines the vector relative degree (*p*_1_, …, *p*_*ℓ*_), which, once again, is well-defined if

$$\begin{array}{c} D\left(x\right)=[\begin{array}{ccc} L_{g_{1}}L_{f}^{p_{1}-1}h_{1}\left(x\right) & \cdots & L_{g_{m}}L_{f}^{p_{1}-1}h_{1}\left(x\right)\\ \vdots & \ddots & \vdots \\ L_{g_{1}}L_{f}^{p_{\ell }-1}h_{\ell }\left(x\right) & \cdots & L_{g_{m}}L_{f}^{p_{\ell }-1}h_{\ell }\left(x\right) \end{array}] \end{array}$$ (39)

is invertible. If such is the case, then ***D*** is known as the system’s *decoupling matrix*. Even in the case of a non-square matrix which means that the number of outputs and inputs are not equal (*ℓ* ≠ *m*), the pseudo inverse can be taken [55]. Analogous to the case of only one output, this creates *ℓ* decoupled linear systems in *normal form*. Let ***z***_*i*,*j*_ be the *j*-th component of the *i*-th system, the linearized system in Byrnes-Isidori normal form is given by

$$\begin{array}{c} \begin{array}{c} {\dot{z}}_{i,1}=\;{\dot{z}}_{i,2},\;{\dot{z}}_{i,2}={\dot{z}}_{i,3},\;\cdots ,\;{\dot{z}}_{i,p_{i}}=v_{i}\;\;\text{with}\;\;y_{i}=z_{i,1} \end{array} \end{array}$$ (40)

for *i* = 1, …, *ℓ*. As before, this system was obtained from (33), where one can define a chain of integrators as above. Defining

$$\begin{array}{c} \Theta \left(x\right)≔[\begin{array}{c} L_{f}^{p_{1}}h_{1}\left(x\right)\\ \vdots \\ L_{f}^{p_{\ell }}h_{\ell }\left(x\right) \end{array}] \end{array}$$ (41)

and putting together (33) for all outputs gives the control law

$$\begin{array}{c} u\left(x\right)=D^{-1}\left(x\right)\left(-\Theta \left(x\right)+v\right). \end{array}$$ (42)

Note that (40) is also a linear system that is controllable. A standard state feedback law can now be applied as in (28) so that the overall system is stable and can be made to follow a desired path. The state feedback law is applied on ***v***_*i*_ = ***K***_*i*_***z***_*i*_, where $z_{i}=(\begin{array}{ccc} z_{1,i} & \cdots & z_{1,p_{i}} \end{array}{)}^{⊤}$ can be obtained from the derivatives of each output and ***K***_*i*_ is a user-defined vector of gains.
